# Supplementary figures and images for: Addressing persistent challenges in digital image analysis of cancer tissue: resources developed from a hackathon
Source: Mol Oncol. 2025 Feb 10;19(6):1565–81. doi: 10.1002/1878-0261.13783 (PMC12161476; doi:10.1002/1878-0261.13783)

### *Typical image analysis pipeline*

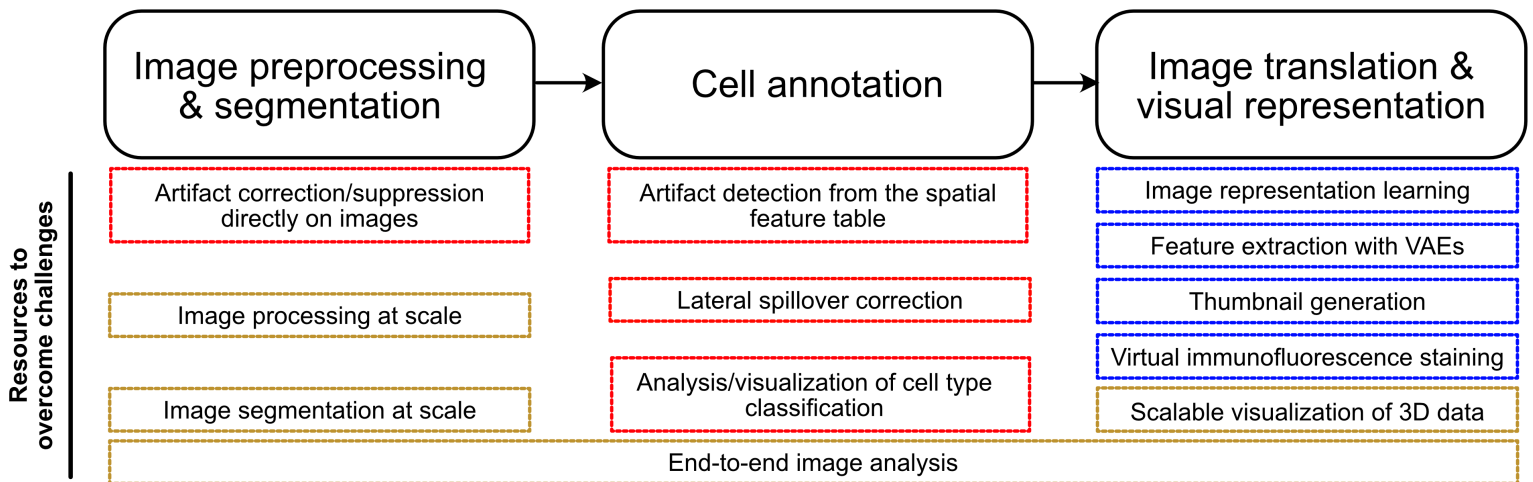

Supplement: Supplementary file 1 — S1. Resources developed within the Image Analysis Hackathon 2022 addressed different aspects of the typical image analysis pipeline for fixed cancer tissues. [file MOL2-19-1565-s009.pdf]

## Before REDSEA

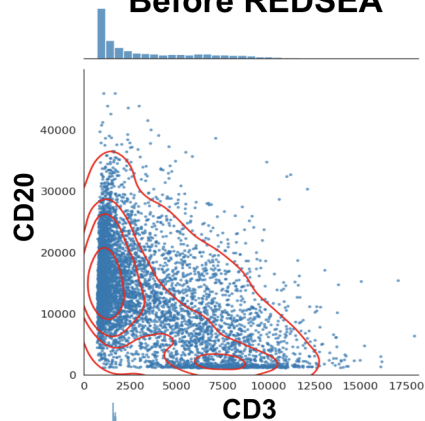

## After modified REDSEA

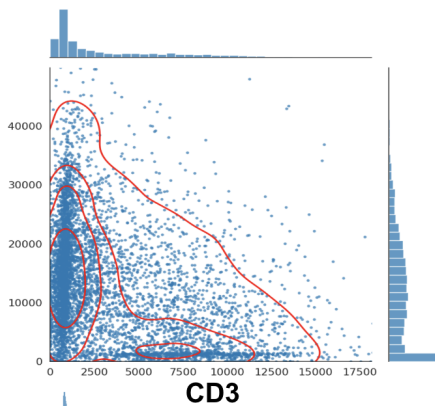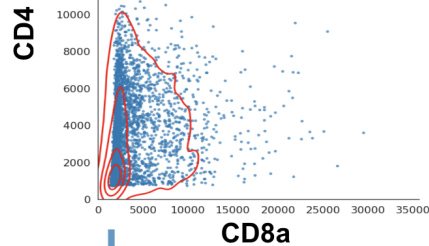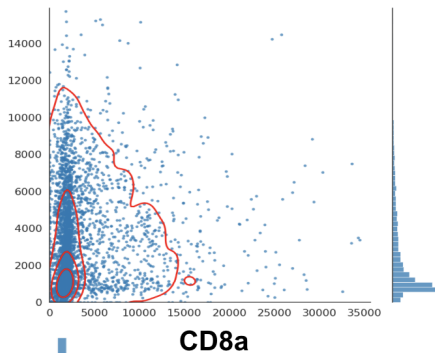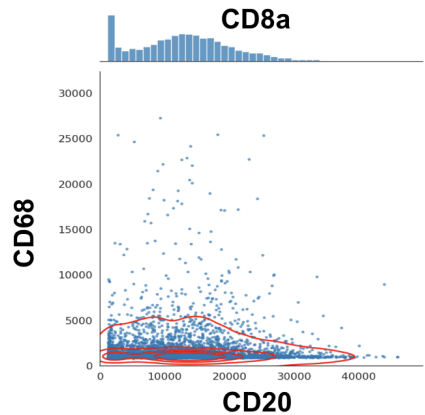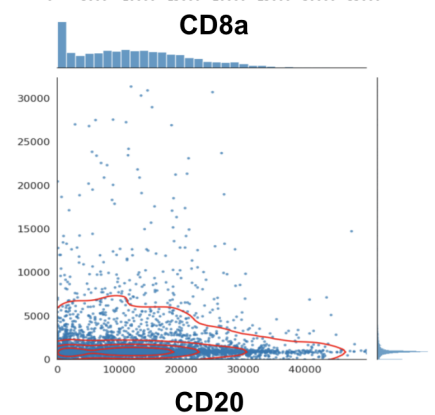

Supplement: Supplementary file 4 — S4. Comparison of cell data before and after application of REDSEA. [file MOL2-19-1565-s006.pdf]

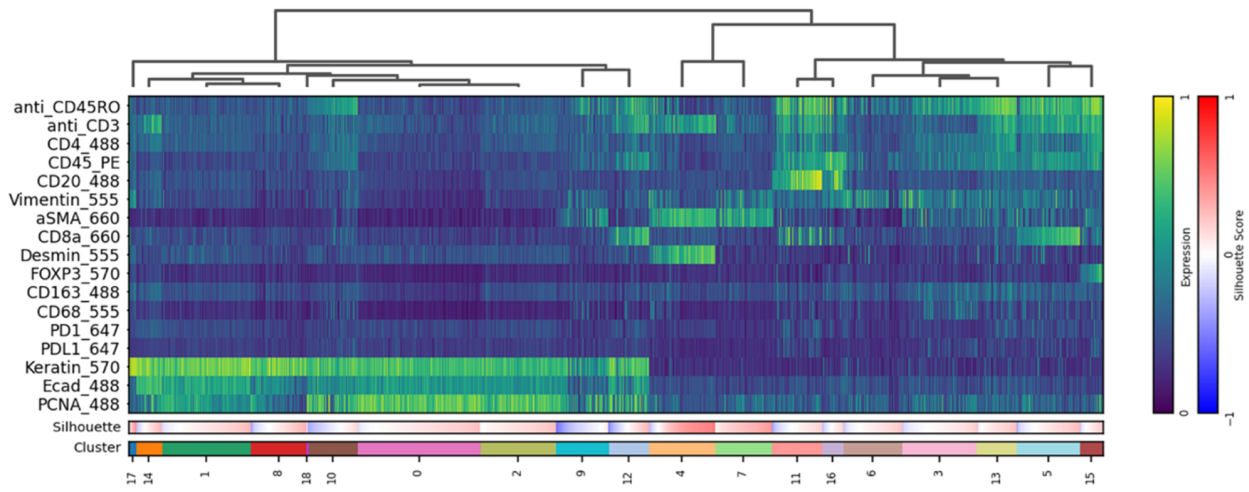

Supplement: Supplementary file 5 — S5. Detailed heatmap for marker expressions at single‐cell resolution with color bars representing silhouette coefficients and cluster memberships. [file MOL2-19-1565-s003.pdf]
