# Supplementary material for: Addressing persistent challenges in digital image analysis of cancer tissue: resources developed from a hackathon
Source: Mol Oncol. 2025 Feb 10;19(6):1565–81. doi: 10.1002/1878-0261.13783 (PMC12161476; doi:10.1002/1878-0261.13783)
Supplement: Supplementary file 2 — S2. Overview of approach to address challenges to automatic artifact detection. [file MOL2-19-1565-s005.pdf]

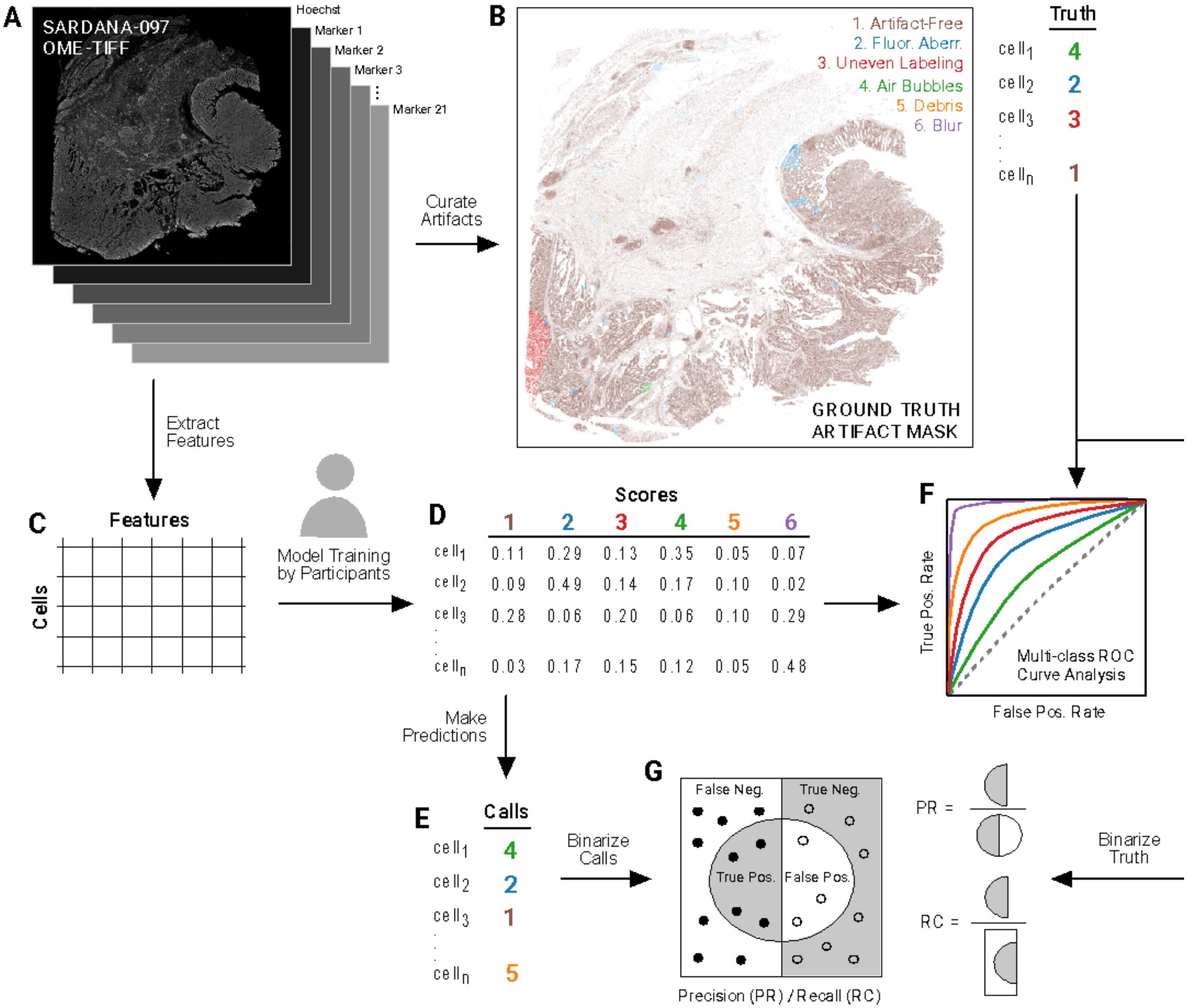
A
SARDANA-097
OME-TIFF
Hoechst
Marker 1
Marker 2
Marker 3
Marker 21
Curate
Artifacts
B
1. Artifact-Free
2. Fluor. Aberr.
3. Uneven Labeling
4. Air Bubbles
5. Debris
6. Blur
GROUND TRUTH
ARTIFACT MASK
Truth
cell1 4
cell2 2
cell3 3
celln 1
Extract
Features
C
Features
Cells
Model Training
by Participants
D
Scores
1 2 3 4 5 6
cell1 0.11 0.29 0.13 0.35 0.05 0.07
cell2 0.09 0.49 0.14 0.17 0.10 0.02
cell3 0.28 0.06 0.20 0.06 0.10 0.29
celln 0.03 0.17 0.15 0.12 0.05 0.48
F
True Pos. Rate
False Pos. Rate
Multi-class ROC
Curve Analysis
Make
Predictions
E
Calls
cell1 4
cell2 2
cell3 1
celln 5
Binarize
Calls
G
False Neg.
True Neg.
True Pos.
False Pos.
Precision (PR) / Recall (RC)
PR =
RC =
Binarize
Truth
