# Supplementary material for: Addressing persistent challenges in digital image analysis of cancer tissue: resources developed from a hackathon
Source: Mol Oncol. 2025 Feb 10;19(6):1565–81. doi: 10.1002/1878-0261.13783 (PMC12161476; doi:10.1002/1878-0261.13783)
Supplement: Supplementary file 3 — S3. Artifact Classifier Performance. [file MOL2-19-1565-s010.pdf]

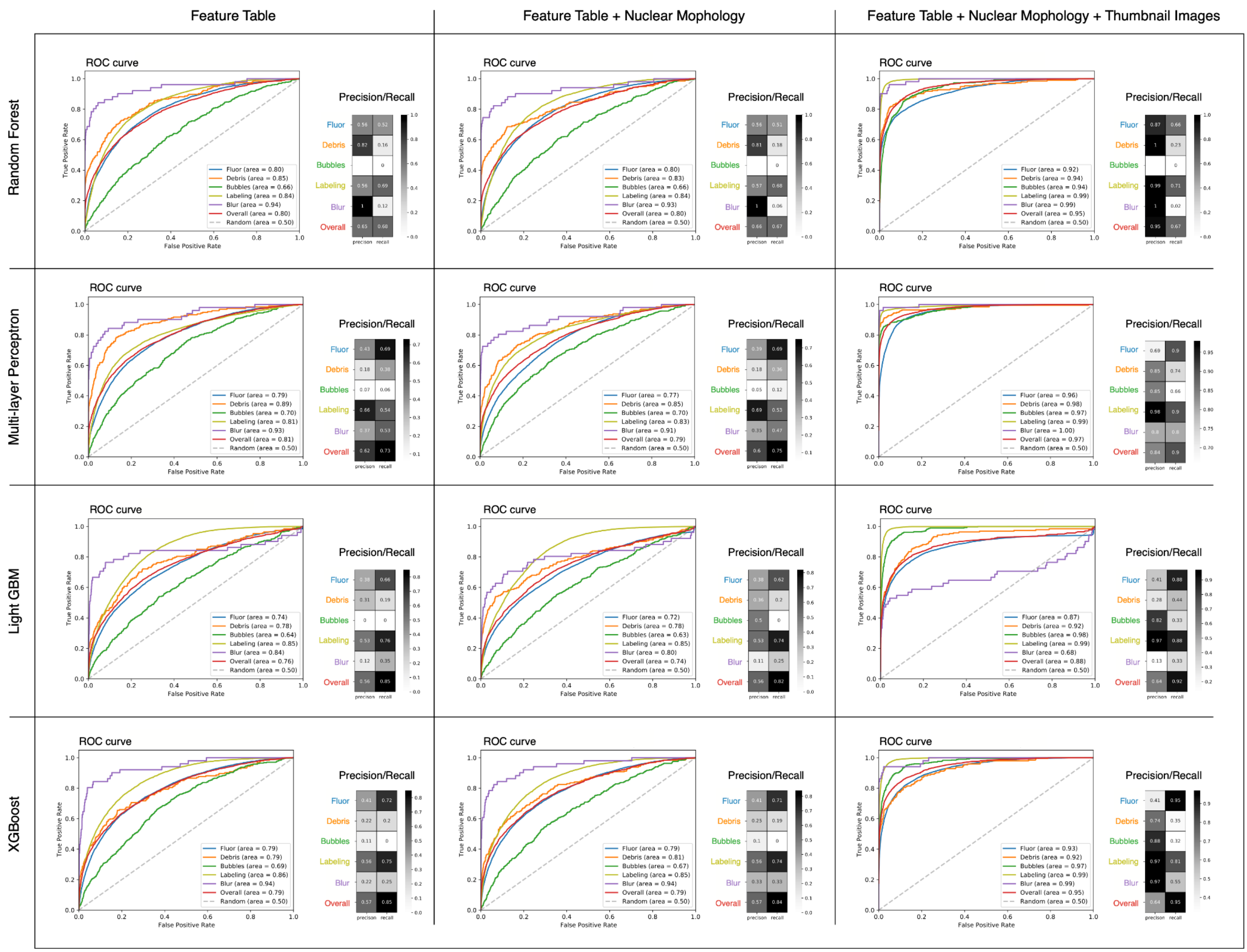

Feature Table
Feature Table + Nuclear Mophology
Feature Table + Nuclear Mophology + Thumbnail Images
Random Forest
Multi-layer Perceptron
Light GBM
XGBoost
ROC curve
Precision/Recall
True Positive Rate
False Positive Rate
Fluor
Debris
Bubbles
Labeling
Blur
Overall
precison
recall
Fluor (area = 0.80)
Debris (area = 0.85)
Bubbles (area = 0.66)
Labeling (area = 0.84)
Blur (area = 0.94)
Overall (area = 0.80)
Random (area = 0.50)
Fluor (area = 0.80)
Debris (area = 0.83)
Bubbles (area = 0.66)
Labeling (area = 0.84)
Blur (area = 0.93)
Overall (area = 0.80)
Random (area = 0.50)
Fluor (area = 0.92)
Debris (area = 0.94)
Bubbles (area = 0.94)
Labeling (area = 0.99)
Blur (area = 0.99)
Overall (area = 0.95)
Random (area = 0.50)
Fluor (area = 0.79)
Debris (area = 0.89)
Bubbles (area = 0.70)
Labeling (area = 0.81)
Blur (area = 0.93)
Overall (area = 0.81)
Random (area = 0.50)
Fluor (area = 0.77)
Debris (area = 0.85)
Bubbles (area = 0.70)
Labeling (area = 0.83)
Blur (area = 0.91)
Overall (area = 0.79)
Random (area = 0.50)
Fluor (area = 0.96)
Debris (area = 0.98)
Bubbles (area = 0.97)
Labeling (area = 0.99)
Blur (area = 1.00)
Overall (area = 0.97)
Random (area = 0.50)
Fluor (area = 0.74)
Debris (area = 0.78)
Bubbles (area = 0.64)
Labeling (area = 0.85)
Blur (area = 0.84)
Overall (area = 0.76)
Random (area = 0.50)
Fluor (area = 0.72)
Debris (area = 0.78)
Bubbles (area = 0.63)
Labeling (area = 0.85)
Blur (area = 0.80)
Overall (area = 0.74)
Random (area = 0.50)
Fluor (area = 0.87)
Debris (area = 0.92)
Bubbles (area = 0.98)
Labeling (area = 0.99)
Blur (area = 0.68)
Overall (area = 0.88)
Random (area = 0.50)
Fluor (area = 0.79)
Debris (area = 0.79)
Bubbles (area = 0.69)
Labeling (area = 0.86)
Blur (area = 0.94)
Overall (area = 0.79)
Random (area = 0.50)
Fluor (area = 0.79)
Debris (area = 0.81)
Bubbles (area = 0.67)
Labeling (area = 0.85)
Blur (area = 0.94)
Overall (area = 0.79)
Random (area = 0.50)
Fluor (area = 0.93)
Debris (area = 0.92)
Bubbles (area = 0.97)
Labeling (area = 0.99)
Blur (area = 0.99)
Overall (area = 0.95)
Random (area = 0.50)
