# Supplementary material for: Addressing persistent challenges in digital image analysis of cancer tissue: resources developed from a hackathon
Source: Mol Oncol. 2025 Feb 10;19(6):1565–81. doi: 10.1002/1878-0261.13783 (PMC12161476; doi:10.1002/1878-0261.13783)
Supplement: Supplementary file 6 — S6. Details of the hackathon, Criteria for team presentations of hackathon performance, List of code repositories (web links), List of abbreviations and acronyms. [file MOL2-19-1565-s002.pdf]

Supplementary Text

Details of the hackathon

Prior to execution of the hackathon, the organizing committee solicited summaries of specific challenges to the image processing and analysis pipeline with potential solutions to these challenges as something that could be addressed within a hackathon setting. There were eleven challenges proposed to the pool of potential participants, who were from labs affiliated with one of the contributing NIH-funded consortia (CSBC, PS-ON, HTAN, HuBMaP, etc.) in addition to numerous external participants who had heard about the hackathon through social media posts or by word of mouth from consortia-affiliated laboratories. There were 85 registrants who expressed their interest in participating in one or more of the proposed challenges but only 32 individuals actually participated in the hackathon, in addition to the “champions” who were involved in the development of the challenge topics (51, in total); the majority of nonparticipating registrants were from outside the US and many of the remaining registrants who did not participate in the hackathon were undergraduate students with no direct affiliation with any consortium-member laboratory.

The hackathon itself was held virtually via Zoom on February 15–18, 2022 with ancillary communication via dedicated Slack channels. Each team was coordinated by their respective champion with regular check-ins to discuss the distribution of tasks and assess progress. On the final day, each team presented a summary of the challenge and their novel contributions toward addressing it and the teams were assessed for a predetermined set of criteria (see below) to award modest prizes donated by sponsors. Three judges (senior investigators from member consortium laboratories) assessed all the presentations and provided scores and feedback to each team. After the completion of the hackathon, work on some of the challenges has continued and some have resulted in separate publications. The challenge champions and other contributors were requested to provide written descriptions of their efforts, which formed the basis for the main text. Contributors of written work and figures are included as named authors on this manuscript, and the complete list of contributors and hackathon participants is provided in Supplementary Table 1.

Criteria for team presentations of hackathon performance

The goal of the presentations is to highlight the progress made within the hackathon toward addressing the specific challenge. The following criteria will be used in assessing the presentations:

- ***Summary of challenge***: Were the problems sufficiently explained? Good balance of succinctness and thoroughness? (10 pts)
- ***Approach***: What options were considered? How many different approaches were tried? How were the outcomes tested/validated? (25 pts)
- ***Outcomes***: What was the primary advancement the team contributed? Was a reasonable solution found or was an approach effectively applied to a new dataset? If not, how much progress was made? Can any approaches be ruled out as unlikely to be effective? Any other useful outcomes? (25 pts)
- ***Next steps***: What work remains to be done? How can the broader community use what was done? (20 pts)
- ***Teamwork***: How did the team leverage the skills of all team members? (10 pts)
- ***Duration***: Was the presentation within the allotted time? (–1 pt/sec past time limit)
- ***Style***: Enthusiasm, audience engagement, visuals, etc. (10 pts)

(Max score was 100)

Code Repositories

Challenges to identifying and classifying cell types

Automatic artifact detection from the spatial feature table:
https://doi.org/10.5281/zenodo.10055947

Artifact correction/suppression directly on images:
https://doi.org/10.5281/zenodo.10056097

Lateral spillover correction using REDSEA:
https://doi.org/10.5281/zenodo.10056002

Python version of original REDSEA (Matlab) code:
https://github.com/labsyspharm/redseapy

Analysis of cell type classification:
https://doi.org/10.5281/zenodo.10056004

Image representation learning

Feature extraction with VAEs:
https://doi.org/10.5281/zenodo.10056000

Thumbnail generation:
https://doi.org/10.5281/zenodo.10056009

Virtual immunofluorescence staining:
https://doi.org/10.5281/zenodo.10055950

Image processing at scale

Deploying image segmentation at scale:
https://doi.org/10.5281/zenodo.10056006

End-to-end image analysis with Galaxy:
https://doi.org/10.5281/zenodo.10056011

Scalable Visualization of 3D data using Neuroglancer:
https://doi.org/10.5281/zenodo.10056036

Additional code repositories

Scatter-gl:
https://github.com/PAIR-code/scatter-gl

PALOM:
https://github.com/Yu-AnChen/palom

CyLinter:
https://github.com/labsyspharm/cylinter

Miniature:
https://github.com/adamjtaylor/miniature

Neuroglancer precomputed data:
https://github.com/google/neuroglancer/tree/master/src/neuroglancer/datasource/precomputed

Auto-Minerva:
https://github.com/jmuhlich/auto-minerva

Image data used during hackathon (all challenges)

https://www.synapse.org/Synapse:syn26848022/files/
